# Glia Crosstalk in Neuroinflammatory Diseases

**DOI:** 10.3389/fncel.2020.00209

**Published:** 2020-07-29

**Authors:** Ada Bernaus, Sandra Blanco, Ana Sevilla

**Affiliations:** ^1^Departament de Biologia Cellular, Fisiologia i Immunologia, Facultat de Biologia, Universitat de Barcelona, Barcelona, Spain; ^2^Molecular Mechanisms Program, Instituto de Biología Molecular y Celular del Cáncer, Consejo Superior de Investigaciones Científicas (CSIC), University of Salamanca, Salamanca, Spain; ^3^Instituto de Investigación Biomédica de Salamanca (IBSAL), Hospital Universitario de Salamanca, Salamanca, Spain

**Keywords:** neurodegeneration, astrogliosis, microglia, proteinopathies, Alzheimer disease, Parkinson disease, amyotrophic lateral sclerosis

## Abstract

Neuroinflammation constitutes a fundamental cellular process to signal the loss of brain homeostasis. Glial cells play a central role in orchestrating these neuroinflammation processes in both deleterious and beneficial ways. These cellular responses depend on their intercellular interactions with neurons, astrocytes, the blood–brain barrier (BBB), and infiltrated T cells in the central nervous system (CNS). However, this intercellular crosstalk seems to be activated by specific stimuli for each different neurological scenario. This review summarizes key studies linking neuroinflammation with certain neurodegenerative diseases such as Alzheimer disease (AD), Parkinson disease (PD), and amyotrophic lateral sclerosis (ALS) and for the development of better therapeutic strategies based on immunomodulation.

## Immune Responses in Neurological Disorders

**Figure 1:**
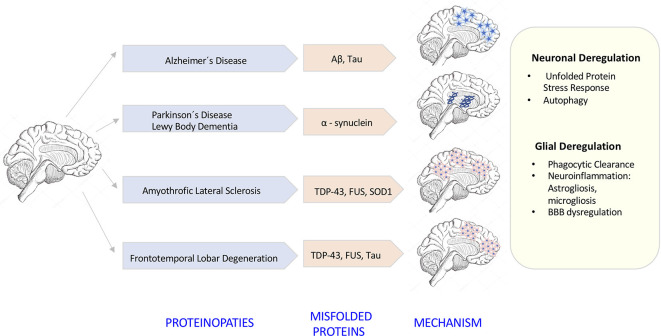
Neurodegenerative diseases induced by proteinopathies. Specific gene mutations induce proteins to take abnormal structural foldings forming small oligomeric or large fibrillary aggregates. These aggregates precipitate at the intraneuronal and extraneuronal space suffering posttranslational modifications that impair its clearance inducing neuroinflammatory responses.

The degeneration of the central nervous system (CNS) is characterized by chronic progressive loss of the structure and neuronal activity, resulting in functional and mental impairments (Campbell et al., 1999). The incidence of neurodegeneration increases in mid to late adult life. As the population ages, there is a clear need for further investigation in the causes of neurodegeneration. So far, although causative agents of neurodegeneration have yet to be identified, the recent evidence shows an accumulation of misfolded proteins self-aggregated at specific parts of the brain, which are linked with significant inflammatory processes and increased oxidative stress (Figure 1). The deregulation of protein clearance mechanisms both at the intracellular (neuronal autophagy) and intercellular levels (interaction among neurons, astrocytes, microglia, and T cells) could be the cause of the neural tissue degeneration.

As we can see, neurodegeneration involves a neuroinflammatory component where both innate and adaptive immune responses play an important role. While the innate immune system components represent the first line of defense against invading pathogens or damage-associated molecules (DAMs), the adaptive immune system components constitute specialized leukocyte populations such as B and T lymphocytes, which are capable of generating specialized antigen receptors to interact with microbial pathogens.

Several lines of evidence have shown that, in the presence of DAMs or infection, one of the first line of defense of the innate immune response is the activation of pattern recognition receptors (PRRs) located mainly within the CNS, in the microglia cells. These receptors are capable of recognizing patterns expressed by both invading pathogens and virus [pathogen-associate-molecular-patterns (PAMPs)] and damage-associated molecular patterns (DAMPs). Among these PRRs we have, the Toll-like receptors (TLRs) and C-type lectin receptors, both located on the membrane surface and the inflammasome components such as the RING-I–like receptors and nucleotide oligomerization domain receptors or NOD-like receptors (NLRs) located and activated inside the cytoplasm.

Engagement of these PRRs triggers downstream signaling pathways that lead to the production of proinflammatory cytokines. Some of these cytokines are produced in their precursor form, which need to be matured by the multimolecular intracellular signaling complex called the inflammasome component, in order to become functionally active (Heneka et al., 2018). Although there are several inflammasomes in the CNS implicated in the progression of neurodegenerative diseases such as NLRP1, NLRP2, NLRC4, and AIM2 (for review see Heneka et al., 2018), we will only highlight the function of the nucleotide-binding oligomerization domain, leucine-rich repeat, and pyrin domain-containing 3 (NLRP3) as the key innate immune sensor for danger signals, because it is the most commonly studied inflammasome within the CNS.

Because both *in vitro* studies and animal models have shown that many proteinopathies stimulate the innate as well as the adaptive immune responses, much work has been conducted to elucidate the role of microglia, astrocytes, and T-cell activation in these disorders to develop better therapeutic strategies focused on immunomodulation. In this review, we will compile the latest evidences regarding the accumulation of these misfolded proteins and the activation of the immune response, which involves several intercellular interactions, involving crosstalks of microglia with neurons, astrocytes, the blood–brain barrier (BBB), and T cells, which eventually infiltrate the CNS parenchyma.

## Microglia Functions in The CNS

Microglia are the resident macrophages of the CNS, which represent about 5% to 12% of total CNS cells in the healthy brain and the spinal cord (SC). Microglia cells derived from myeloid precursor’s cells from the yolk sac during embryogenesis that colonize the developing brain during embryogenesis, where they proliferate and persist throughout the individual’s lifetime (Ginhoux et al., 2010). During this early stage, microglia presents an active phagocytic capability pruning the excess of synapsis formed, which is necessary for normal brain development (Paolicelli et al., 2011).

Quite recently, microglia have been studied at the single-cell level through scRNAseq and mass cytometry, showing a very interesting phenotypical heterogeneity across different regions of the mouse brain (Ko et al., 2013; Tasic et al., 2016). Both techniques have confirmed the existence of higher numbers of microglia cells in the cortical regions (72%) than in the cerebellum and SC (19%) of mice. This distribution is also conserved in both early embryonic and adult human brains (Herculano-Houzel, 2012).

Besides the heterogeneity in the cell number at the different brain areas, it is remarkable that these differences also involve their capability to divide, size, density of receptors expressed, and ramification patterns (Tan et al., 2020). Based on their ramification patterns, microglia cells have been graded as ramified (numerous thin processes distributed in a radial manner), primed (thickened processes with reduced secondary branching and increased proliferation rate and polarity), reactive (thickened processes and barely branching), or ameboid (rounded soma with no branching; Tay et al., 2017).

**Figure 2:**
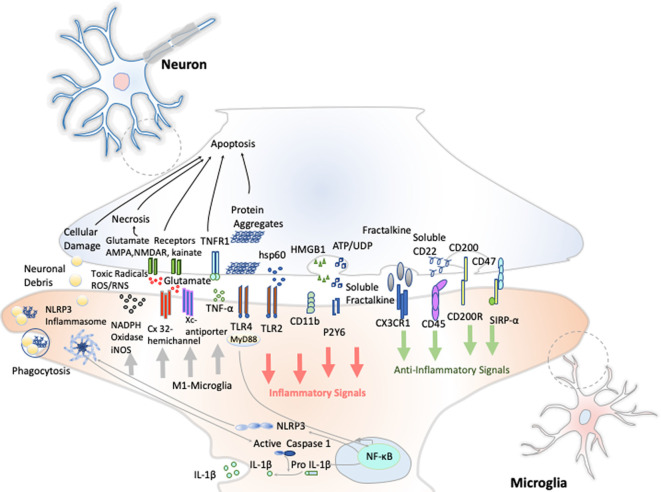
Neuron–microglia crosstalk. An amplification of neuron–microglia interactions is shown in green arrow signals. Homeostatic neurons actively produce anti-inflammatory signals aimed at inhibiting the acquisition of the inflammatory M1-like phenotype by microglial cells. During neurodegenerative disorders or after nervous system injury, cellular mediators associated with damage are activated (salmon arrows) triggering the M1 microglia state that releases reactive species of oxygen and nitrogen (ROS and RNS), glutamate and TNF-α (gray arrows) Importantly, an oxidative environment favors further oxidation and self-aggregation of proteins in damaged neurons, which promotes neuronal death that subsequently stimulates TLRs and the NLRP3 inflammasome in microglia enhancing the production of IL-1β.

Resting microglia presents a ramified morphology and elongated motile cytoplasmic processes that are constantly surveying the microenvironment and actively interact with other neighboring cells, such as neuronal cell bodies, astrocytes, and oligodendrocytes. Indeed, communication between neurons and microglia is crucial for optimal regulation of behavior and brain physiology (Figure 2). One of the ways of such communication is driven by the fractalkine receptor (CX3CL1) secreted from neurons and its microglial target (CX3CR1) receptor (Paolicelli et al., 2014). The activation of this G*α*i-coupled seven transmembrane domain receptor modulates several intracellular signaling pathways (PLC, PI3K, and ERK), and the recruitment of transcription factors [nuclear factor κB (NF-κB), cAMP response element-binding (CREB)] activating specific gene expression programs (Sheridan and Murphy, 2013). Fractalkine/CX3CR1 signaling pathway modulates microglial activation in a broad spectrum. It participates in microglia migration and dynamic surveillance of the brain parenchyma, survival of developing neurons, their maturation, synapse pruning, plasticity, and brain functional connectivity, having a final impact in learning and memory capability (Paolicelli et al., 2014). Disruption of this interaction, according to several transgenic animal models of neuropathology, including Alzheimer disease (AD), Parkinson disease (PD), amyotrophic lateral sclerosis (ALS), and stroke, leads to increased production of proinflammatory molecules. Concretely, interleukin 1β (IL-1β) and reactive oxygen species (ROS) trigger a massive cell death (Sheridan and Murphy, 2013). However, paradoxically in two AD mouse models, CX3CR1 deficiency decreases microglia activation and production of proinflammatory molecules such as IL-1β, tumor necrosis factor-α (TNF-α), and monocyte chemoattractant protein 1 (MCP-1 or CCL2), increasing its phagocytic activity and helping the reduction of β-amyloid protein accumulation (Lee et al., 2010). Thus, these studies show that neuroprotective and neurotoxic functions of this signaling pathway are dependent on the pathological context, disease stage, and microglial activation stimuli from the CNS microenvironment.

Another receptor studied in detail is the glycoprotein CD200 widely found in cell membranes of neurons, astrocytes, and oligodendrocytes (Koning et al., 2009). CD200 interacts with microglial CD200R, to keep also microglial cells in its inactivated or resting state (Hoek et al., 2000). In addition, another inhibitory receptor/molecule complex is the integrin CD47, which communicates a “do not eat me signal” to microglial C172a by down-regulating phagocytosis in synaptic pruning during the development and by increasing the synthesis of transforming growth factor β (TGF-β) through the recruitment of tyrosine-protein phosphatases SHP-1 and SHP-2 (Lehrman et al., 2018). Therefore, the disruption of any of these inhibitory interactions drives microglia activation.

Besides all these neuron–microglial communications through receptor–ligand interactions, neurons can also release immune-related soluble factors such as neurotrophins, neuropeptides, neurotransmitters, anti-inflammatory cytokines, and chemokines that bind to cognate receptors on microglia, modulating cell functions and tissue homeostasis maintenance. Notably, TGF-β, which is expressed in glial cells and neurons, has been recognized as a key regulator for microglia differentiation, promoting a more alike transcription profile and surface structure of adult microglia (Butovsky et al., 2014). The binding of TGF-β to its receptor induces the activation of a receptor kinase, subsequent phosphorylation, and activation of SMAD proteins. SMAD pathway, activated by TGF-β receptors, has been considered as one of the main signal transduction pathways responsible for its neuroprotective effects (Abutbul et al., 2012). Another soluble factor released by neurons after injury is CD22. This ligand binds CD45, a leukocyte common antigen, constitutively expressed in microglia at moderate levels. The binding of these two molecules leads to the negative regulation of the Src/p44/42 MAPK cascade inhibiting microglia as well (Tan et al., 2000).

Remarkably, in the presence of infectious agents, the large family of microglia TLRs (TLR1–TLR9) specialized in PAMPs including compounds derived from bacteria, virus, or fungi are the ones that get activated (for review see Fiebich et al., 2018). These receptors recognize molecules such as lipopolysaccharides (LPSs), flagellin, or double-stranded RNA, although they also have been shown to be activated in both human and mouse brains within different neurodegenerative diseases (Zolezzi et al., 2019). Importantly, except for TLR3, which initiates signaling *via* the Toll/IL-1R domain-containing adaptor-inducing interferon-β (IFN-β; TRIF) adapter, the rest of the TLRs signaling pathways are dependent on myeloid differentiation primary response 88 (MyD88)-associated protein. Notably, TLR4 can activate signaling *via* both TRIF and MyD88. The MyD88-dependent pathway triggers the recruitment of tumor necrosis factor–receptor associated factor 6 (TRAF6) and members of the IL-1R–associated kinases (IRAK) family. The activation of TRAF6 permits the translocation of NF-κB into the nucleus, activating a transcriptional program for the production of different proinflammatory mediators such as cytokines, inducible nitric oxide synthase (iNOS), and cyclooxygenase 2 (COX-2; Kawai and Akira, 2010). On the contrary, the MyD88-independent pathway is more associated with the induction of IFN-β-inducible genes (Yamamoto et al., 2003).

Toll-like receptors also participate actively in the activation of both canonical and noncanonical NLRP3 inflammasome. Different studies have shown that canonical NLRP3 inflammasome activation requires a transcriptional step first regulated by innate immune signaling, mediated primarily by MyD88 and/or cytokine receptors, such as the TNF receptor, which in turn activate pro–IL-1β and NLRP3 transcription *via* NF-κB activation (Bauernfeind et al., 2009). The second step results in NLRP3 inflammasome oligomerization, leading to caspase-1 activation and, in turn, IL-1β and IL-18 processing and release (Faustin et al., 2007). Various stimuli associated with infection, β-amyloid fibers, extracellular osmolarity, ATP, PH alterations, and degradation of extracellular matrix components, increase in potassium efflux, ROS, cathepsin activation, and deubiquitylation, can promote NLRP3 inflammasome oligomerization and activation. Additionally, caspase-1 activation promotes also, independently from IL-1β maturation, pyroptosis, a key defense mechanism against microbial infections, which blocks the replication of intracellular pathogens by cytoplasmic swelling and promotes phagocytosis of surviving bacteria (Aachoui et al., 2013).

Besides canonical NLRP3 inflammasome activation, a noncanonical caspase-11–dependent NLRP3 activation has been characterized (Kayagaki et al., 2011). In particular, Gram-negative bacteria activate TLR4-MyD88 and TRIF pathways triggering nuclear translocation of NF-κB, which in turn promotes the transcription of IL-1β, IL-18, and NLRP3, as well as caspase-11 gene. In particular, once activated, caspase-11 induces pyroptosis through cleavage of gasdermin, as well as high mobility group box-1 (HMGB1) and IL-1α release, and promotes IL-1β processing and release through activation of the NLRP3–ASC–caspase-1 pathway.

These different NLRP3 activation processes occur independently. However, caspase-11 enhances the canonical caspase-1 processing and IL-1β/IL-18 production in the presence of specific stimuli (e.g., cholerae toxin or *Escherichia coli*, Kayagaki et al., 2011). In this setting, further research needs to be done to clarify the molecular mechanisms underlying the interplay between caspase-1 and caspase-11 in promoting the canonical and/or noncanonical NLRP3 inflammasome activation.

In summary, microglia activation seems to be a highly regulated biological process; however, the molecular mechanisms underlying their activation are not yet fully understood. Having a very simplistic view of the process, we can summarize that after a CNS injury or infection, there is an initial inflammatory response mediated by M1-like microglia. This early activation plays a dual beneficial role for the brain involving microbicide activity against most of the pathogens and phagocytic activity for the clearance of cellular debris necessary for later repair of lesions. After this early activation, M1-like microglia can be transformed into M2-like microglia, a beneficial state that contributes to the attenuation of the inflammation stage previously induced by M1-like microglia, and, simultaneously, produce neurotrophic factors to repair the affected tissue (Shechter et al., 2013). Or on the other hand, M1-like microglia can go into an uncontrolled activation for long periods of time, which triggers chronic inflammation releasing neurotoxic factors, such as TNF-α, IL-6, IL-1β, IL1-α, nitric oxide (NO), hydrogen peroxide, superoxide anion, chemokines such as RANTES and MCP-1, proteolytic enzymes, and glutamate, thus ending in neuronal loss over time (Kettenmann et al., 2011).

Because of the diverse morphology of microglia and the cytokines released, more microglia categories have been identified than the simple M1 (proinflammatory) and M2 (anti-inflammatory), especially in a number of disease states that we will describe below (Deczkowska et al., 2018).

## Astroglia Functions in The CNS

Astrocytes are not easily defined because of the notable heterogeneity exhibited on both their morphology and function. They constitute the principal cell type of the CNS, and the term encompasses all those cells that do not belong to the other classified cell types: neurons, microglia, and oligodendrocytes. This diverse morphology allows astrocytes to carry out a wide range of functions, which increase as more research is done in the field. They serve as metabolic support for neurons, establish synapses and modulate the ones that have already been established, and are the principal modulator of the brain’s homeostasis (Morita et al., 2019). Concurrently, astrocytes are able to repair injuries in the brain, respond to insults, and build a continuous crosstalk with microglia, thus playing an important role in the brain’s immune system. Finally, astrocytes are also components of the BBB, which determine the circulating molecules that will reach the CNS (Kery et al., 2020).

The most classical morphological division was the one proposed by Ramón y Cajal in 1909. He divided them into protoplasmic (highly ramified) and fibrous astrocytes (with longer prolongations). As more studies are being developed, multiple evidences are pointing out that the morphology of astrocytes varies along with their age state, brain layer location, and brain region as we have previously described for the microglia (Garwood et al., 2017).

**Figure 3:**
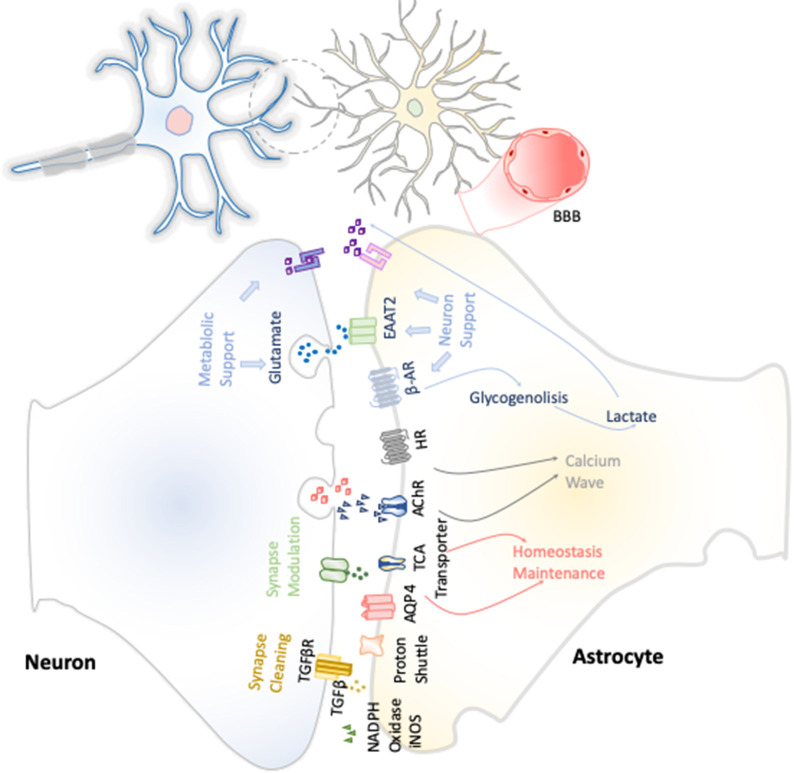
Neuron–astrocyte crosstalk. These neuron–astrocyte interactions are focused on providing neuronal metabolic support, maintenance of the homeostasis through synaptic cleaning, and synaptic modulation.

The first function attributed to astrocytes was their metabolic support of neurons (Figure 3). Astrocytes constitute the only energy reservoir neurons can resort to, as, by themselves, they cannot store glycogen (Pfeiffer-Guglielmi et al., 2003). In this way, in the event of exceptional high neuronal function, astrocytes release noradrenaline and, *via* β-adrenergic receptors, activate glycogenolysis as short-term energy buffer mechanism for the stimulated neurons (Dienel and Cruz, 2016).

Astrocytes are responsible for maintaining the homeostasis in the whole brain environment, in every aspect: ion concentration, pH, and neurotransmitter concentrations in synaptic interstitial fluid, ensuring the correct environment is key for maintaining neural function and synapses. This is done through diverse membrane transporters such as aquaporin 4, different proton pumps, bicarbonate transporters, and monocarboxylic acid transporter. Furthermore, they express transporters of neurotransmitters such as glutamate, γ-aminobutyric acid (GABA), and glycine (Sofroniew and Vinters, 2010). Currently, multiple evidences have been gathered on the direct role of astrocytes in synaptic transmission. This fact has been baptized as tripartite synapse, which comprises not only both neurons presynaptic and postsynaptic membranes, but also the astrocytes’ contribution (Perea et al., 2009). Because the neurotransmitters discharged from the presynaptic membrane, such as glutamate, acetylcholine, noradrenaline, and histamine, are close enough to the astrocyte surface, they easily interact with their receptors. This triggers the initiation of signaling pathways that would vary, depending on the signal emitted by the presynaptic axon, but often leads to calcium waves by G protein–coupled receptor activation, which can potentially be transmitted to neighboring astrocytes through their gap junctions (Kofuji and Araque, 2020). The calcium concentration increase is usually translated into the release of gliotransmitters and neuroactive molecules that ensure basic synaptic activity and modulate the existing neural synapses.

Thus, astroglia contributes to synaptic processes in a direct and indirect manner uptaking and releasing neurotransmitters, being highly remarkable their role in glutamate uptake and release. Astrocytes present glutamate receptors and excitatory amino acid transporters (EAATs) to sense the neural state, and according to it, they can modify their metabolic pathways for suiting the needs of neurons at every moment (Lee and Pow, 2010). In addition, they are also key in the process of new synapse formation and proper synaptic transmission, because these cells also conduct elimination of aberrant or superfluous synapses by either direct phagocytosis or *via* the production of TGF-β (Diniz et al., 2017). Moreover, astrocytes promote oligodendrocyte progenitor cell differentiation and enhance myelination through the production of leukemia inhibitory factor-like protein, neuregulin, gamma secretase, ciliary neurotrophic factor, and neurotrophin-3, among others. They promote the process on each stage from oligodendrocyte progenitor cells to their differentiation and myelinization (Ishibashi et al., 2006).

## Microglia and Astrocyte Crosstalk in Neuroinflammation

The relationship between microglia and astrocytes is still unclear. However, as neuroinflammation takes a more important role in the study of a wide range of brain pathologies and affectations, it is becoming clearer that both cell types engage in a constant fine and intimate crosstalk. This crosstalk is key in resting, activated, and aged state, but it is only beginning to be understood (Liu et al., 2012; von Bernhardi et al., 2015; Kery et al., 2020).

Microglia are the cells of the brain’s innate immune system. They are the first cells that dynamically respond to proinflammatory stimuli and, when the situation is resolved, counteract for inflammation, driving astrocytes to a beneficial profile by the secretion of IL-10. This cytokine triggers astrocyte’s TGF-β production, which is a neuroprotective molecule and restrains inflammation, reinforcing the noninflammatory microglia M2 phenotype. Indeed, TGF-β protects synapses in AD from the deleterious effect of β-amyloid deposition in murine cultures (Diniz et al., 2017). However, if the situation is not successfully resolved or under chronic inflammation, microglia cells increase in number switching to the reactive phenotype, known as microgliosis, and deliver complement component 1q protein (C1q), TNF-α, and IL-1β into the brain environment. These molecules are sensed by astrocytes as potent inflammation inducers and react, producing astrogliosis. This abnormal increase in the number of astrocytes cause secretion of even more proinflammatory stimuli and therefore initiates the reactive loop. Remarkably, for most patients with neurodegenerative disease, reactive astrocytes are ubiquitous in the CNS tissues (Li et al., 2019).

One of the most proinflammatory and perhaps pernicious cytokine produced by both microglia and astrocytes is the TNF-α. Among the various effects, it negatively affects oligodendrocytes and produces demyelization. For example, in mixed glial cultures, when exposed to LPS, microglia releases important amounts of TNF-α, which lead to systemic activation of the immune response. TNF-α not only affects the immune response and cytokine production, but also boosts α-amino-3-hydroxy-5-methyl-4-isoxazolepropionic acid receptor (AMPA receptor) production while reducing that of GABA receptors, thus enhancing the activator synapsis and causing an extreme neural activity (Stellwagen et al., 2005).

Prostaglandin D_2_ (PGD_2_) is an important inflammatory mediator produced by reactive microglia, through their contact with the receptors DP1 and DP2 receptors expressed in astrocyte membranes. When the receptors sense PGD_2_, they swap to a proinflammatory phenotype, glial fibrillary acidic protein (GFPA) production is increased, blood flow is locally increased, antigen presentation is enhanced through the expression of the intercellular adhesion molecule 1 in astrocytes, and chemotactic factors are finally released (Mohri et al., 2006).

Moreover, NO production by microglia yields to an increase of glycolytic enzymes on astrocytes, leading to ROS production and hypoxia inducible factor 1α release (Iizumi et al., 2016). When ROS is produced by astrocytes forming the BBB, vasodilatation is triggered, favoring the recruitment of monocytes (Prajeeth et al., 2017). Furthermore, in response to NO astrocyte production, microglia enhance their IL-1β production, which is received as a proinflammatory cytokine by astrocytes, which will further increase NO production (Sudo et al., 2015). As a result of all the interactions, both cell types acquire a dangerous extreme inflammatory state, known as astrogliosis and microgliosis, characterized by the up-regulation of proinflammatory molecules IL-1β, IL-6, TNF-α, and reactive species NO and ROS, creating an unsuited environment for neurons leading to synapse loss and neuron death (Sedel et al., 2004). This environment of inflammatory cytokines such as TNF-α and IL-1β has also a direct effect on the BBB endothelial cells up-regulating the expression of several cell surface molecules specific for T-cell infiltration. The down-regulation of the tight junctions on the endothelial cells of the BBB allows the entrance of the components of the adaptive immune response. T cells infiltrate in the parenchyma of the CNS through different mechanisms. For instance, the expression of vascular cell adhesion molecule 1 on endothelial cells of the BBB favors the entrance of activated lymphocytes into the inflamed CNS by direct interaction with surface α4-integrins (Vajkoczy et al., 2001).The surface-activated leukocyte cell adhesion molecule induced on endothelial cells during the inflammation of the BBB binds to CD6 expressed on T cells, allowing their entrance into the brain parenchyma (Cayrol et al., 2008). Besides the expression of specific receptors for T cells, endothelial cells also secrete chemokines such as CXCL9, CXCL10, CXCL11 (Kivisakk et al., 2002), CCL19, CCL21, and MCP-1 (Engelhardt, 2006), which constitute important chemoattractants for the successful recruitment of CD4^+^ T cells through the BBB during neurodegenerative diseases. The restimulation of autoreactive CD4^+^ T cells with inflammatory phenotypes in the CNS parenchyma would result in a strong production of inflammatory cytokines, which act directly or indirectly in microglia and infiltrated macrophages, exacerbating their M1-like inflammatory properties. Therefore, the infiltration of inflammatory T cells in the CNS parenchyma after an initial microglia-mediated neuroinflammation results in a positive feedback, spreading, and perpetuating neuroinflammatory processes involved in neurodegenerative diseases.

## Neuroinflammation in Alzheimer’s Disease

AD is responsible for most cases of dementia and affects a great part of the older population. Unfortunately, even though it is on continuous search, neither a cure nor an efficient diagnosis method for its early stages has been found. For these reasons, new approaches are arising with the objective of finding new ways to ameliorate or prevent AD.

**Figure 4:**
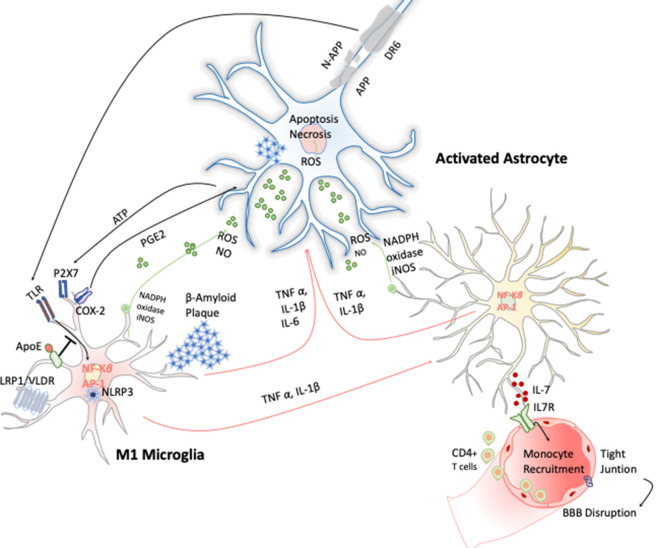
Inflammatory response in Alzheimer disease (AD). Amyloid precursor protein (APP) is cleaved producing amyloid-β peptide that aggregates. These aggregates activate microglia cells through TLRs and RAGE changing the transcriptional program of the microglia cells activating the expression of transcription factors like AP-1 and NF-κB, which in turn trigger the production of reactive oxygen species (ROS) and inflammatory cytokines. These cytokines amplify the proinflammatory state by the stimulation of the astrocytes that together act directly on the neuronal population (cholinergic, glutamatergic, and GABAergic neurons) inducing neuronal cell death. Apoptosis of neurons results in release of ATP, which further activates microglia through purinergic P2X7 receptor, entering in an autostimulatory loop inducing T-cell infiltration.

AD is a chronic neurodegenerative condition characterized by the deposition of aberrant protein β-amyloid, the formation of neurofibrillary tangles produced after the hyperphosphorylation of tau. The symptoms are progressive memory decline, altered behavior, and loss of social–emotional function. The β-amyloid plaque is produced because of the sequential cleavage of the amyloid precursor protein (APP), first by the γ-secretase and then by the β-site APP cleaving enzyme 1 (BACE-1; Hampel et al., 2020; Figure 4).The β-amyloid aggregates, in particular their oligomeric or fibrillar forms, act as DAMPs activating the NLRP3 inflammasome in microglia, among other effects (Halle et al., 2008), as well as in astrocytes (Couturier et al., 2016), enhancing the release of mature IL-1 β. This fact has been evidenced in brains of AD patients with a substantial increase in the expression of NLRP3 canonical signaling pathway through caspase-1 activation (Saresella et al., 2016).

As more data are being compiled about the aged microglia activity, it is not surprising that neuroinflammation plays an important role in this neurodegenerative disease (Hampel et al., 2020). In fact, under systemic inflammation conditions, the NLRP3 inflammasome signaling pathway has been shown to be a key mediator of detrimental microglial effects during aging (Tejera et al., 2019). Indeed, age itself favors inflammation in the brain, without the need of previous infections or immune insults. Along with age, GFPA expression levels in astrocytes also increase. The increase of GFPA induces the acquisition of a phenotype that could resemblance that of reactive astrocytes (Rozovsky et al., 1998). This fact is also supported by transcriptomic data that show that age drives changes of the whole gene expression profile of astroglia toward a proinflammatory and senesce phenotype (Clarke et al., 2018). Moreover, astrocytes that come from the brains of elder mice exhibit a lower capacity of response and produce lower levels of the cytokine IL-10, which exhibits a putative anti-inflammatory neuroprotective effect, favoring its chronic and continuous activation (Norden et al., 2016).

Astrocytes switch from atrophic phenotype in the early stages of the disease to a hypertrophic one in the later stages. This change could be triggered by the accumulation of amyloid plaques. In fact, the deposition of β-amyloid by itself could explain astrogliosis (Serrano-Pozo et al., 2011). Notably, microglia cells exert a putative beneficial function at the beginning, favoring the phagocytosis of the β-amyloid plates. Clearance can be performed through receptors such as CD36, the low-density lipoprotein receptor-related protein (LRP1), and triggering receptor expressed on myeloid cells 2 (TREM2), which needs the participation of apolipoprotein E provided by the astrocytes. However, when the clearance is not sufficient, the proinflammatory profile chronifies, and then both microglia and astrocyte acquire a new deleterious profile (for review see Ries and Sastre, 2016). This aberrant inflammatory phenotype is mainly characterized by the production of superoxide ions, TNF-α, and IL-1β, which act as both autocrine and paracrine stimuli on the other glial cell types. TNF-α favors tau phosphorylation while also triggering the NF-κB pathway (Lattke et al., 2017), thus leading to the production of iNOS; S100B, which leads to tauopathy; and BACE-1 production (Chen et al., 2012). The cytokines produced, which are IL-6, TNF-α, and IL-18, have detrimental effects on neural progenitors causing their death and accelerating the neurodegeneration already caused by the fibrous depositions. The general chronic swollen state gives rise to metabolic alterations and increases the secretion of NOS and ROS species involved in axonal and synaptic damage, β-amyloid nitration, neuronal apoptosis, and final cognitive impairment (Bronzuoli et al., 2016).

In the same way, astrocytes entering in an autostimulative circle disrupt the tripartite synapses and the correct maintenance of the synaptic environment, decreasing the blood flow. This alteration of the neuronal vascular unit could heavily affect neurons’ oxygen supply (Kisler et al., 2017). Damaged and dying neurons release a multitude of signals, including cytokines, miRNAs, and apoptotic bodies that remain, capable by themselves of triggering the astrocytic and microglia immune response in a vicious circle. Moreover, the deposition and accumulation of β-amyloid and fibrinogen plaques do also favor the astrocytic activation, synapse loss, and memory alteration as well (Bronzuoli et al., 2016).

Additionally, a number of postmortem studies have confirmed the presence of T cells in the brains of AD patients. Recently, it has been demonstrated the presence of CD3^+^ extravascular T cells, which correlate with tau pathology in the brains of AD patients, but not with the number of β-amyloid plaques (Merlini et al., 2018). Interestingly, polymorphisms in genes associated with antigen presentation to T cells were identified as susceptibility loci for AD (Lambert et al., 2013), lending further support to the potential relevance of T cells in AD.

Studies in the murine model AD susceptible mice, known as 5XFAD mice that express human APP and presenilin (PSEN1) transgenes with a total of five AD-linked mutations, have shown accelerated accumulation of β-amyloid plaques and exacerbated neuroinflammation even though their adaptive immune system is genetically ablated (*Rag2^−/−^/Il2rγ^−/−^*-5XFAD mice). Thus, the findings suggest a protective role for adaptive immunity in the diseased brain. Microglial cells present increased cytokine production and reduced phagocytic capacity in these mice. Moreover, wild-type bone marrow transplantation into these immunodeficient mice resulted in a 47% reduction in β-amyloid plaque volume (Marsh et al., 2016). On the contrary, another study using APP/PS1 mice that express human transgenes for mutations in APP and PSEN1, crossed with lymphocyte-deficient *Rag2^−/−^* mice, showed 25% to 30% less β-amyloid plaque pathology than the APP/PS1 mice at 8 months of age (Spani et al., 2015).

Because the role of adaptive immunity on AD pathogenesis remains ambiguous, depending on the mouse model used and the progression stage of the pathology, alternative anti-inflammatory approaches are being tested as treatment for AD. Peroxisome proliferator-activated receptor gamma (PPAR-γ) agonists have been demonstrated to ameliorate the inflammatory state, decreasing NO levels in brain murine primary cultures, as well as proinflammatory cytokine production (Dentesano et al., 2014). At preclinical and clinical levels, the study of cannabinoid receptors and inflammation has shown a negative correlation, leading to the proposal of cannabinoid receptor’s agonists as treatment in AD (Aso et al., 2013).

A more direct approach to inflammation is being studied, and inhibitors of proinflammatory molecules are being tested. For instance, one approach is the inhibition of NLRP3 inflammasome by fenamate, a class of nonsteroidal anti-inflammatory drugs that by attenuating microglia activation reduces the cognitive deficits in two rodent models of AD *in vivo* (Daniels et al., 2016). Moreover, antibodies blocking the proinflammatory cytokines IL-1β (Kitazawa et al., 2011) and TNF-α (Shi et al., 2011) have shown to decrease the tau kinase activity and lessen the deposition of fibrous aggregates, whereas the inhibition of COX-2 and iNOS has shown to ameliorate the disease in both *in vivo* and *in vitro* studies (Scuderi and Steardo, 2013; Scuderi et al., 2014; Gan et al., 2015).

A more indirect onset would be, instead of blocking the immune response, to reprogram it into a beneficial outcome. Following this line of thought, active immunization through the administration of glatiramer acetate or Copaxone, has demonstrated a neuroprotective action in animal models of Alzheimer (Butovsky et al., 2006). Glatiramer acetate decreases the proinflammatory cytokines production through microglia and—lymphocyte interaction promoting microglia phagocytic activity, as well as IL-10 production and secretion (Pul et al., 2012). Another promising immunomodulator is fingolimod or Glienya. When administrated to microglia cells *in vitro*, it reduces TNF-α, IL-6, and IL-1β culture production (Noda et al., 2013). Ethyl pyruvate acts by lowering the levels of microglia-produced HMGB1, a chromosomal protein of nonhistonic nature, which has been also linked with senile plaques (Shin et al., 2015).

Lastly, a target that is now gaining a lot of attention is glycogen synthase kinase-3β (GSK3β), which is implicated in microglial migration and inflammation-induced neurotoxicity through astrocytes. These GSK3β inhibitors such as NP12 or tideglusib reduced efficiently β-amyloid deposition and tau pathology on AD mouse models (Onishi et al., 2011). Unfortunately, they have shown no neuroprotection on clinical trials, but many other small blockers of GSK3β are being developed and tested (Lovestone et al., 2015).

## Neuroinflammation in Parkinson’s Disease

PD is the second most common neurodegenerative disease after AD and is the most common movement disorder. Prominent clinical features are motor symptoms (bradykinesia, tremor, rigidity, and postural instability) besides other non-motor-related symptoms such as olfactory deficits, autonomic dysfunction, depression, cognitive deficits, and sleep disorders. Currently, about 2% of the population older than 60 years is affected. From all the PD diagnosed cases, 15% of people with PD have a family history. Familial cases of PD have been linked to mutations in the LRRK2, PARK7, PINK1, PRKN, or SNCA gene, among others.

Like AD, PD is another proteinopathy. It is characterized by the accumulation and aggregation of misfolded α-synuclein. This protein acts as an intracellular component localized at the presynaptic terminal (Lashuel et al., 2013). α-Synuclein is one of the most prevalent pathological genes altered in familial PD. Generally, the mutated forms of α-synuclein are nitrated or oxidized and aggregated forming the Lewy body structures, which constitute the neuropathological hallmarks used for its diagnostic. This accumulation of misfolded protein α-synuclein in the Lewy bodies is the cause of the final loss of dopaminergic neurons (DA) mainly in the substantia nigra of the midbrain but also in other brain regions (Braak et al., 2003). Postmortem tissue from brains of PD patients have also revealed an increased number of astroglia cells and in particular dystrophic astrocytes (Braak et al., 2007). In this regard, various PD toxin-base mouse models, such as 1-methyl-4-phenyl-1,2,3,6-tetrahydropyridine (MPTP), 6-hydroxydopamine, and rotenone, as well as mutant α-syn transgenic models of PD (M7KO, M83KO and SYNKO), have also demonstrated microgliosis with elevated levels of inflammatory cytokines IL-1β, IL-2, IL-4, IL-6, IFN-γ, and TNF-α, high levels of enzymes related to inflammation such as COX-1, COX-2, and iNOS, and reduced levels of neurotrophins, such as nerve growth factor and brain-derived neurotrophic factor (BDNF; Liu et al., 2019).

**Figure 5:**
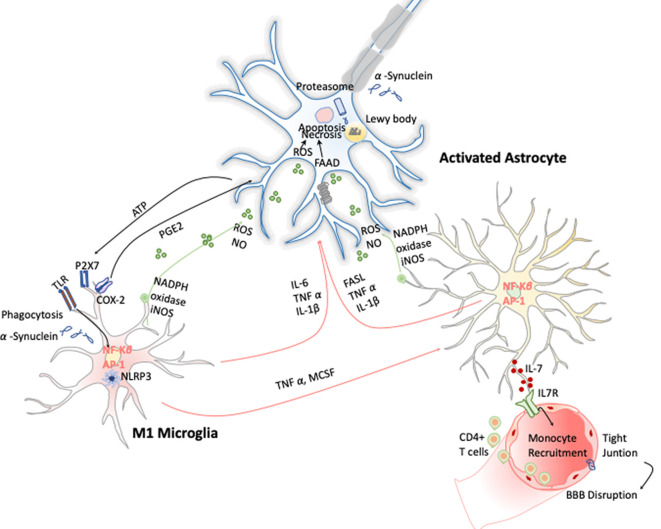
Inflammation in Parkinson disease (PD). The hallmark of this disease is characterized by the presence of intracellular inclusions of α-synuclein aggregates in Lewy bodies. When they are released from neurons, they activate microglia cells through a TLR-independent mechanism. This leads to the activation of NF-κB and production of ROS and proinflammatory cytokines that simultaneously activate the astrocytes and in a combinatorial manner promote neurotoxicity.

These early evidences have helped to study in depth this phenomenon and demonstrate that PD has a clear neuroinflammatory component mainly triggered by the neuronal signals and the release of the α-synuclein aggregates after DA neuronal cell death. Several studies have shown that the release of α-synuclein aggregates induce activation of microglia cells toward M1 phenotype by directly engaging the TLR1/2 heterodimer *via* the MyD88-dependent pathway, which conducts the downstream activation of MAPKs and translocation of NF-κB, p38, and JNK into the nucleus. The translocation of NF-κB into the nucleus results in production and release of proinflammatory cytokines such as TNF-α, IL-1β, and IL-6. These cytokines activate COX-2, iNOS, and NADPH oxidase, leading to the production of NO, ROS, and reactive nitrogen species (RNS; Gao et al., 2008) and inducing reactive astrocytes (Liddelow et al., 2017; Figure 5). Likewise, TLR4 has also been shown to mediate α-syn-dependent activation of microglia, inducing the production of ROS and proinflammatory cytokines, as well as the phagocytic activity (Hughes et al., 2019). Moreover, systemic activation of NLRP3 inflammasome inducing IL-1β production has also been observed by the release of α-synuclein from DA neuronal cell death. Notably, these two observations are highly correlated with motor severity and disease progression in PD patients (Fan et al., 2020). With respect to the noncanonical NLRP3 inflammasome, Furuya et al. (2004) analyzed the immune response in MPTP-injected caspase-11^−/−^ mice and showed a lower microglial activation of IL-1β and NOS in the substantia nigra compared to wild-type mice. Thus, dopaminergic neuronal cell death in the substantia nigra may be mediated in part by the activation of caspase-11 inflammatory signaling cascades (Furuya et al., 2004).

In view of the multiple pathways involved in PD neuroinflammation, a plethora of mechanisms have been studied to either modulate the inflammatory response of microglia or enhance the M2 phenotype, reviewed in Liu et al. (2019). Considering that microglia-derived oxidative stress is believed to bridge α-syn pathogenic alteration and neuroinflammation, many therapeutic approaches have been focused on blocking these receptors. Among them, it is noteworthy to mention the inhibitors of TLRs such as candesartan, cilexetil, and rifampicin; inhibitors of the JAK/STAT signaling pathway or NF-κβ such as α-asarone; inhibitors of the NADPH oxidase such as tanshinone and apocynin to suppress the production of ROS among others, and MCC950, a small-molecule inhibitor of NLRP3 inflammasome that prevents dopaminergic degeneration. Additionally, minocycline, a tetracycline antibiotic that selectively inhibits M1 polarization, has prevented neurodegeneration not only in MPTP mouse models but also has alleviated the clinical signs of early PD in a randomized, double-blind clinical trial (Investigators, 2006).

Another strategy for an efficient therapy could be the use of enhancers to restore the M2 microglia phenotype. In this line, the effects of synthetic analogs of cyclic AMP, vitamin D, PPAR-γ agonists, and iron chelators have been tested (Liu et al., 2019). So far, although these are promising candidates for future clinical trials in PD, their mechanisms are not fully understood and require further investigation.

On the other hand, the participation of the adaptive immune response has also been described in PD. T-cell activation and infiltration of the substantia nigra and striatum have been observed in MPTP models (Chandra et al., 2017).This result is also supported by recent studies carried out with T-cell receptor (TCR) β chain–deficient mice, SCID mice, and recombination-activating-gen-1 (RAG1) knockout (*Rag1*^−/−^) mice, which demonstrated that T-cell deficiency results in a strong attenuation of dopaminergic neurodegeneration in MPTP-induced PD (Brochard et al., 2009). In particular, CD4^+^ T cells infiltrating the substantia nigra during MPTP-induced PD produced high levels of IFN-γ and TNF-α. These two cytokines act synergistically in microglia promoting the inflammatory M1-like phenotype (Barcia et al., 2011).

Recently, genome-wide association studies meta-analysis on data from 12,000 PD patients and 21,000 controls have revealed changes in DNA methylation and expression levels on several gene variants, including PARK16/1q32, GPNMB, and STX1B [International Parkinson’s Disease Genomics Consortium (IPDGC) (2011)]. These findings have opened the possibility that the onset or progression of PD could be associated with epigenetic variations such as DNA methylation or histone modifications. The initial hypothesis suggested that reduction of methylation levels of PD-related genes, such as SNCA (α-synuclein), could help with an increase in their expression levels, leading to the abnormal accumulation of α-synuclein and the degeneration of DA (Feng et al., 2015). However, although the regulation of the SNCA promoter by DNA methylation has been thoroughly investigated, many findings are inconclusive and sometimes contradictory (Guhathakurta et al., 2017). On the other hand, data proving that microglia can be activated to the M1 phenotype simply by reduction of the H3K27me3 demethylase Jmjd3 and the fact that Jmjd3 seems to be essential for M2 microglia polarization suggest that this demethylase indeed has a pivotal role in the switch of microglia phenotypes that may contribute to the immune pathogenesis of PD (Tang et al., 2014).

Based on these data, epigenetic-based therapies are arising. The most promising of these therapies is the approach of using histone deacetylase inhibitors (HDAC inhibitors). This approach is based on the hypothesis that accumulations of α-synuclein may “mask” acetylation sites on histones proteins, thus causing deregulation in the dynamic control of gene transcription. One possible gene candidate to target is Sirt1, a class III HDAC that acts *via* broad deacetylation at various histone residues at loci including transcription factors, epigenetic enzymes, and NF-κB gene, responsible for up-regulating gene products that control cell survival (Singh et al., 2017).This observation has opened a new approach for testing if neurodegeneration could be attenuated by HDAC inhibitors. Among the HDAC inhibitors, valproate (VPA) was considered to be the most promising drug for the treatment of PD, because VPA not only increases histone acetylation and reduces the expression of inflammatory factors, but also induces expression of BDNF and glial cell-derived neurotrophic factor (GDNF) for neuroprotection (Harrison and Dexter, 2013). However, even though it was a promising candidate, clinical trials with VPA did not alter PD features in PD patients (Nutt et al., 1979). Conversely, a recent nonrandomized phase I clinical trial with phenylbutyrate has successfully shown removal of α-syn from the brain into the bloodstream (ClinicalTrials.gov identifier: NCT02046434). Mechanistically, this promising HDAC inhibitor can up-regulate the protein deglycase (DJ-1). DJ-1 acts as a versatile prosurvival factor in DA activating different protective mechanisms such as regulation of mitochondria, antioxidative stress response, and up-regulating total intracellular glutathione in response to a diverse range of PD related insults (Repici and Giorgini, 2019).

## Neuroinflammation in Amyotrophic Lateral Sclerosis

Amyotrophic Lateral Sclerosis (ALS) is a fatal adult paralytic disorder caused by the death of upper and/or low motor neurons (MNs) in the motor cortex, SC, and brain. The symptoms may vary from one patient to another, but generally they present disable weakness, spasticity, and loss of control of the voluntary muscles (Valori et al., 2014). Even though it is the most common adult-onset MN disease, with an incidence of 1–3 cases in 100,000 people worldwide (Valori et al., 2014), no effective treatment has been yet elucidated. ALS patients live with the terrible prognosis of 2–5 years of life expectancy, which is only extended by a few months under the treatment of rizulone, the only approved drug until recently for the disease. Nowadays, a second drug has been authorized, edaravone, and its combined administration have entailed a mild improvement, but the result is yet far from satisfactory (Scott, 2017).

The vast majority of ALS cases, approximately the 90%, are sporadic, vs. the 10% of them that are familiar cases (Pasinelli and Brown, 2006). The most common mutation affects the SOD-1 gene, which was the first targeted gene in ALS. More recently, TDP-43 and OPTN have also been considered (Liu et al., 2018; Lutz, 2018). Mechanistically, the OPTN mutation identified in various ALS patients promotes inflammation *via* NF-κB as already shown in the SOD-1 mouse models as well (Frakes et al., 2014). This NF-κB activation, although observed in astrocytes, is much more pronounced in microglia, which leads to the secretion of IL-1β, IL-6, and TNF-α in both *in vitro* cultures and in mouse models, giving rise to elevate nerve cell death (Liu et al., 2018). Moreover, TDP-43 mutations seem to be able to instigate the canonical *via* of NLRP3 inflammasome activation in microglia enhancing the production of IL-1β and IL-18, through caspase-1 activation. All these effects result in an enhancement of the proinflammatory signaling that is detrimental to MNs (Zhao et al., 2015). Alternatively, Kang et al. (2003) investigated the role of knocking down the noncanonical NLPR3 inflammasome pathway generating the caspase-11^−/−^ SOD G93A transgenic mice. Although mice exhibited lower caspase-1 and caspase-3, as well as lower levels of IL-1β, the inhibition of caspase-11 was not sufficient to prevent the disease onset and progression (Kang et al., 2003).

**Figure 6:**
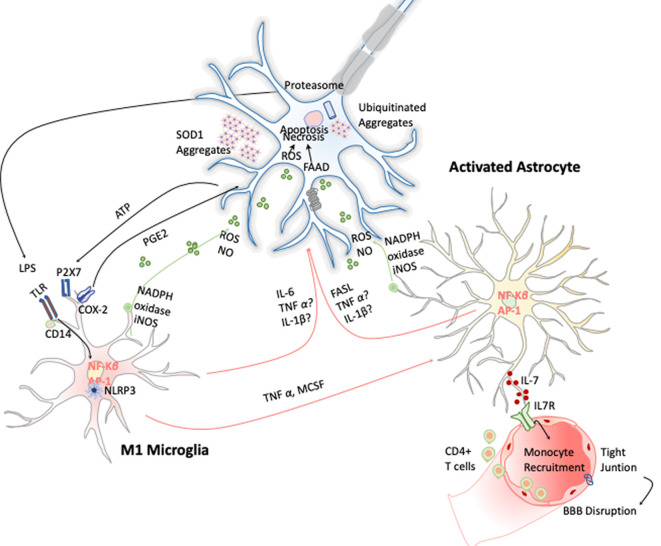
Inflammation in amyotrophic lateral sclerosis (ALS). Toxic aggregates of SOD1, TDP-43, or FUS can induce inflammatory responses and activate microglia *via* TLR2 and CD14. This leads to the expression of the transcription factor NF-κB and AP1, which trigger the production of proinflammatory cytokines and apoptosis inducers such as TNF-α and FASL. Dying motor neurons (MNs) release ATP, which further activates the purinergic receptor P2X7 expressed in microglia.

Microgliosis has revealed to be a typical hallmark on ALS disease in both ALS patients and animal models. Increased expression of inflammatory markers such as CD45, CD11b, IBA-1, and CD68 have been observed in most affected brain areas. Positron emission tomography imaging studies of ALS patients further confirm this microglial activation (Zürcher et al., 2015). Additionally, TREM 2 is also overexpressed and correlates negatively with patient’s prognosis, being a potential biomarker for the disease (Cady et al., 2014). Activated microglia can induce oxidative stress *via* ROS and NO and proinflammatory cytokines such as TNF-α, IL-6, and IL-1β, which induce MN cell death (Garbuzova-Davis et al., 2018). These cytokines activate astrocytes, which also affect ALS onset and development by two different approaches. On the one hand, astrocytes secrete toxic and proinflammatory molecules capable of causing MN death by itself; and on the other, the disregard of their functions as neuronal supporters also compromises MN survival (Valori et al., 2014). In this line, several observations validate the hypothesis of astrogliosis effecting ALS. Up-regulation of GFPA, SOD-1 protein aggregation in astrocytic soma, and increase of COX-2 and iNOS expression are some examples (Norden et al., 2016; Figure 6). Moreover, it has been observed that the introduction of mutant SOD-1 astrocyte precursors in the SC of wild-type mice triggered MNs’ degeneration symptoms (Papadeas et al., 2011). Supporting these data, mutant SOD-1 astrocytes in coculture with MNs also decreased the MN survival (Phatnani et al., 2013).

Besides the activation of the innate immune response, there are evidences indicating that the adaptive immune system also plays an important role regulating microglia phenotypes in the progression of ALS. In the SOD1 mutant mouse (SOD1mt), a model of familial ALS, lymphocyte infiltration into the CNS has been observed, most prominently at later stages of the disease (Alexianu et al., 2001). SOD1mt transgenic mice crossed with *Rag2* ^−/−^ mice (deficient for B and T lymphocytes) or with a mouse model lacking T cells (TCRβ^−/−^ mice) developed an accelerated progression to the symptomatic stage of ALS (Beers et al., 2008; Chiu et al., 2008). Further experiments performed with SOD1mt crossed with CD4^−/−^ mice (deficient of CD4^+^ T lymphocytes) recapitulated the same phenotype as in *Rag2* ^−/−^ mice, thus attributing the neuroprotective role to CD4^+^ T cells, but not to CD8^+^ T cells or B cells (Beers et al., 2008). In fact, when lacking CD4^+^ T cells, ALS mouse microglial cells display decreased levels of trophic factors such as insulin like growth factor 1 (IGF-1), GDNF, and BDNF; increased levels of proinflammatory cytokines such as IL-6 and TNF-α; and elevated levels of NADPH-oxidase 2, which is known to enhance microglial release of ROS (Beers et al., 2008; Chiu et al., 2008). All these data suggest that CD4^+^ T cells may play a regulatory role on microglial cells during ALS progression, providing supportive neuroprotection by favoring the acquisition of the M2-like phenotype by microglial cells, although further research is still needed to develop future therapeutic strategies for ALS in this line.

What has been further explored is the purpose of astrocytes as therapeutic targets in ALS analyzing diverse approaches through multiple candidates. Glutamate clearance could become a putative solution, because the astrocyte-specific glutamate transporter (EAAT2) is being identified as defective in human patients and ALS mouse models, and glutamate accumulation triggers neurodegeneration (Kim et al., 2011). Indeed, mutant SOD-1 astrocytes present an increased production of D-serine, capable to activate *N*-methyl-D-aspartate enhancing neuronal cell death caused by glutamate accumulation (Sasabe et al., 2012). Similarly, neuroinflammation can be targeted directly, switching the focus onto proinflammatory mediators. PGD_2_, IFN-γ, and TGF-β produced and secreted from astrocytes have been proposed as inducers for MN death (Phatnani et al., 2013). Finally, another strategy would be targeting free radicals and astrocytic mitochondrial dysfunction because ROS and NO production is up-regulated in astrogliosis. ALS mouse models display defective mitochondria, and human astrocytes expressing mutant SOD-1 exhibit NOX-2 activation and ROS hyperproduction (Cassina et al., 2008). ROS could damage lipid and neuron’s membrane proteins, such as IGF-1 receptor, which switch the redox state of intracellular proteins and exacerbate the glutamate accumulation as previously mentioned (Wu et al., 2006). Thus, proposing improving mitochondrial activity or antioxidant molecules as a potential neuroprotective treatment could open new possibilities for treatment.

More recently, because microglial NLRP3 inflammasome activation is emerging as a key player in neuroinflammation during neurodegeneration, new therapeutic approaches are looking for NLRP3 inhibitors (Deora et al., 2020). In this regard, the NLRP3 inflammasome inhibitor, Cyclo (His-Pro), constitutes a potential candidate for ALS treatment and other misfolding diseases (Grottelli et al., 2019), although further research is still needed.

In summary, all these studies have led to the conclusion that ALS is a noncell autonomous multifactorial disease that implies different cell types and in which cell-to-cell interaction plays a central role, not only in its development, but also on its onset (Valori et al., 2014). Supporting this theory, robust activation of microglia and astrocytes has been observed in postmortem ALS human tissue and animal models (Tam et al., 2019), as well as CD4^+^ T cells in both SOD1 mutant mice (Alexianu et al., 2001) and in ALS human brains (Kawamata et al., 1992).

## Concluding Remarks and Perspectives

Based on what we have described above, activation of the neuroinflammation processes requires the orchestration of transcriptional programs to secrete a diverse repertoire of cytokines and neurotransmitters, which in turn activate innate and adaptive immune response. These cytokines and neurotransmitters served as messengers for the communication between microglia, astrocytes, and neurons. However, our knowledge on how all of these cytoquines and cell types regulate and trigger the activation of each other is still limited. What would be extremely informative is to determine the timing and secretion levels of these cytokines coupled with the expression of receptors by single-cell molecule analysis in all three populations in correlation with the stimuli. Understanding microglia and astrocyte immunomodulatory mechanisms will be key because on one side, they served as amplifiers for the immune response, but on the other side they are capable to elegantly fine-tune the magnitude of this signal inhibiting microglia activation. Therefore, some intriguing questions are deciphering the inhibitory effect of activated astrocytes on microglial activities, as well as the mechanisms that make microglia cells change from their M1 proinflammatory state to their M2 anti-inflammatory state. Unfortunately, certain evidences show that there is a “point of no return” in the neuroinflammation process where the activated astrocytes would be unable to inhibit proinflammatory signals from the microglia, such as the production of NO (von Bernhardi and Eugenin, 2004). In this term, the degree of the inflammatory stimuli seems to be primordial.

Since the timing, stage, and severity of the disease are critical determinants of microglial phenotypes, the first step toward development of new therapeutic strategies would be the identification, through single-cell transcriptomics, proteomics, and functional studies, of stage- and time specific-molecular signatures associated with each microglial–neuron–astrocyte phenotype tailored to the different stages of each neurodegenerative pathology. These would serve as biomarkers, as well as offer a plethora of molecular targets for future research.

Additionally, data from knockout mice, *in vitro* studies and clinical neuroimaging, will significantly enhance the development of pharmacological immunoregulatory strategies to attenuate inflammatory M1 monocytes/macrophages or CD4^+^ T cells and to strengthen the function of M2 monocytes/macrophages or regulatory T cells specific for CNS antigens involved in neurodegenerative disorders.

Other important factors to take into account in these proteinopathies are sex and age. CNS disorders such as ALS (affect male > female) and AD (affect females > males) have been shown to have a sex bias. Moreover, we also need to consider that with age there is a decline in physiologic protective processes, vital for maintaining the body homeostasis. If we sum up the fact that there is a lower phagocytic capability to clean the insult (either protein aggregates generated in many of these neurodegenerative diseases or virus or pathogens) with the fact that immune cells are in general more reactive during aging, this favors a persistent inflammatory state or chronic inflammation that can contribute directly or indirectly to the etiology of the most common neurodegenerative diseases. Therefore, future microglia-based therapeutic strategies should be developed with caution and should address the sex bias and aged microglia physiology seen in disorders of the CNS.

Finally, immunomodulation of microglia may be coupled with inhibition of other signaling pathways in different cell types (astrocytes or neurons) and such combinatorial therapies may enhance functional outcomes. Thus, unraveling these crosstalk mechanisms that inhibit uncontrolled inflammation (Kery et al., 2020) would bring a more comprehensive picture of the whole neuroinflammatory process for the development of future treatments preventing neurodegeneration.

In this line and considering the limitations of using animal models for neurodegenerative diseases, which in most cases poorly recapitulate the complexity of the human disease, future research should be focused on the generation of human models. In this regard, human brain organoids from induced pluripotent stem cells derived into all CNS cell components will provide a tridimensional and more physiological and humanized environment where we could monitor human microglia interactions with other brain cells offering a relevant human model to study brain function and pathologies. The generation of these *in vitro* reliable and easily reproducible human stem cell-based models will significantly enhance our understanding of the pathogenesis of neurodegenerative diseases and could be extremely useful for finding and testing, at different disease stages, efficient therapies at the preclinical stage.

## Author Contributions

AS: manuscript design and drafting. AB, SB, and AS: literature search, manuscript editing and review. All authors read and approved the final manuscript.

## Conflict of Interest

The authors declare that the research was conducted in the absence of any commercial or financial relationships that could be construed as a potential conflict of interest.

The handling Editor declared a shared affiliation, though no other collaboration with several of the authors (AS and AB).
